# Toward Smart Soil Sensing in v4.0 Agriculture: A New Single-Shape Sensor for Capacitive Moisture and Salinity Measurements

**DOI:** 10.3390/s20236867

**Published:** 2020-11-30

**Authors:** Christophe Escriba, Eli Gabriel Aviña Bravo, Julien Roux, Jean-Yves Fourniols, Michel Contardo, Pascal Acco, Georges Soto-Romero

**Affiliations:** 1 Laboratory for Analysis and Architecture of Systems, LAAS, University of Toulouse, F-31077 Toulouse, France; eli-gabriel.avina-bravo@tnpconsultants.com (E.G.A.B.); roux.julien31@gmail.com (J.R.); fourniols@laas.fr (J.-Y.F.); pacco@laas.fr (P.A.); gsotorom@laas.fr (G.S.-R.); 2 TeleCommunications Services & Distribution, TCSD, 82000 Montauban, France; m.contardo@tcsd.fr

**Keywords:** smart sensing, connected agriculture, capacitive bi-sensor, soil moisture and salinity

## Abstract

Modern agriculture imposes the need for better knowledge of the soil moisture content to rationalize the amount of water needed to irrigate farmlands. In this context, since current technological solutions do not correspond to the cost or use criteria, this paper presents a design for a new original capacitive bi-functional sensor to measure soil moisture and salinity. In this paper, we outline the design stages from simulation to finished elements of the optimal design to deployment in the fields, considering the mechanical integration constraints necessary for industrialization. The measurement electronics were developed based on the sensor’s electric model to obtain a double measurement. An on-site (field lot) measurement program was then carried out to validate the system’s good performance in real-time. Finally, this performance was matched with that of leading commercially available sensors on the market. This work demonstrates that, after deployment of the sensors, the overall system makes it possible to obtain a precise image of cultivated soil’s hydric condition, with the best response time.

## 1. Introduction

In modern and ecological agriculture, knowledge of the soil’s hydric condition has become an economic factor of significant importance for the water supply to crops. Accordingly, knowledge of moisture content and salinity is essential to the development of new irrigation systems. To meet this need, one of the most widely employed measurement methods is tensiometers [1], but they do not measure moisture. They measure the matrix potential, which gives information on the soil’s state that can be used to oversee the irrigation. This type of sensor has two main drawbacks: the response time of several hours, during which water is being supplied [2], as well as a phenomenon known as uncoupling [3]. These failings make it problematic to check the quantity of water provided naturally (rain) or artificially (irrigation). Not only is it impossible to check the soil in real-time, while these sensors are very sensitive, they often cease to work when the soil is too wet or too dry. In the latter event it becomes necessary to reinstall sensors, which can be prejudicial to large farm operations.

Consequently, a new capacitive measuring technology has been developed to modernize future agricultural installations. This capacitive measuring principle is selected to measure moisture level in the soil [4,5,6]. This type of sensor’s main advantage is the response time of less than a minute, allowing the soil’s hydric condition to be monitored in close to real-time [7]. Existing solutions [8,9,10] include sensors based on a small-sized (<1 mm) detection cell, which limits the volume of soil that can be tested. However, their complex structures [10,11,12,13] do not make them easy to use and require some time (several months) to restructure the soil, which can be harmful to farmers. Some commercial sensors (Enviroscan, Meteor) are available but their cost limits their deployment and do not permit a reliable coverage of the fields; others (Divine) cannot be implemented for a growing season and provide just a portable measurement.

Since alternative technologies to measure moisture exist, let us examine the radiofrequency waves method [14,15]. This approach does not allow for a double measurement to evaluate soil salinity at the same time. Our solution exploits a sensor structure’s capacitive properties to respond to this need as it makes it possible to measure salinity and moisture [16,17]. Salinity sensing is based on the measurement of the electrical conductivity of the soil. The nutrients in the ground modify its conductivity as measured by an electronic system [18,19]. For this reason, subject to a certain range of frequencies, soil capacity depends not only on moisture but also on its ionic composition [20]. Thus, by taking one moisture measurement followed by a salinity measure, the same sensor can obtain both parameters.

‘The multiplication of measurement points makes it possible to reach areas sufficiently representative of the soil’s hydric condition to allow measurement on the scale of a massive agricultural operation. Several systems have already been created, but either their cost largely limits their deployment [21], or the existing system is technologically limited for large-scale use [22].

In response to these technological barriers, our innovation is based on the design of a new generation of affordable capacitive sensors compatible with deployment demands on a large scale. Section 2.2 presents an elementary sensor design and its optimization. Section 2.2.1 offers a modification of the shape factor of the electrodes to optimize measurement parameters. Section 2.4.3 describes the sensor’s electric model, while Section 2.5.2 presents the design of onboard electronics for the associated measurement. Finally, Section 2.6 outlines the results of on-site performance tests on actual agricultural operations.

## 2. Materials and Methodology

### 2.1. Calibrating Samples

To reproduce field conditions in the laboratory, we sample soil in culture. We use soil composed of 60% clay, 30% compost, and 10% limestone, which is sifted with a 1 mm mesh to obtain homogeneous soil. The soil is heated to eliminate all water contained and is weighed before and after the heating phase. If the weight does not change, the soil is dry. If so, a new phase begins and the soil is separated into calibrated samples.

A known quantity of water relative to the sample’s weight is added to the samples to obtain a precise moisture range.

(1) $$\%RH=\frac{m_{water}}{m_{soil}}*100\;\{\begin{array}{c} \%H\;is\;the\;soil\;humidity\;in\;\%\\ m_{water}\;is\;the\;water^{\prime }\;mass\;in\;g\\ m_{soil}\;is\;the\;soil^{\prime }slass\;in\;g \end{array}$$

For example, for a soil’s mass of 2 kg, if we want the moisture to be around 30%, we add 600 g of water. The sample is mixed manually to make it homogeneous.

A known quantity of fertilizer is added to the water to increase its salinity, but the samples receive the same amount of water.

**Figure 1:**
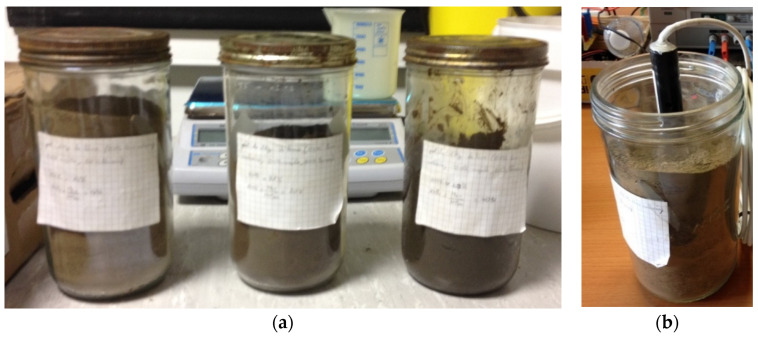
Calibrated samples in jars (**a**) and the measurements (**b**).

The samples are stocked in airtight jars (Figure 1) to avoid any evaporation which could modify them.

To take the measurement, we buried the sensor in a jar. Since the jar is made of glass and covered with its lid (Figure 1a), the measurement is not disturbed by the electromagnetic field’s external humidity, and the measurement is correct (Figure 1b). We use an impedance-meter that can sweep the measurement frequency to obtain the results presented in this paper.

### 2.2. Basic Design Approach

**Figure 2:**
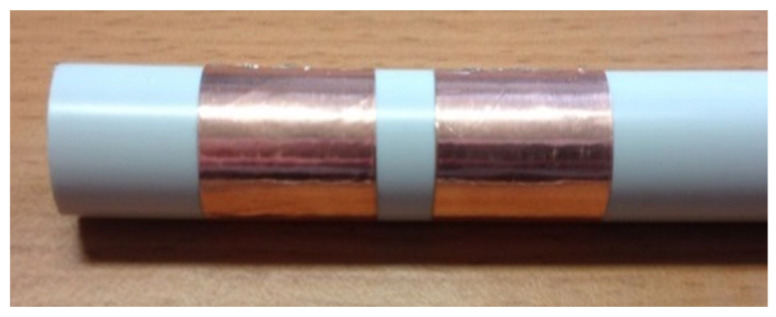
First design sensor.

The transducer’s design is based on the same principle as an elementary shape capacitor (Figure 2). The structure rests on two electrodes separated by a dielectric image of the soil. A cylindrical shape is preferred for this type of transducer in order to facilitate insertion into the ground. Copper electrodes are fixed to the cylinder, as shown in Figure 2. Since the thickness of the electrodes is less than 1/10 mm, the inter-electrode *C_electrodes_* capacity is negligible.

For mechanical solidity reasons, a 40 mm diameter is selected to demonstrate the initial transducer’s feasibility. Its advantages are:

1. Easy insertion into the soil. An auger with a standard diameter is used to make a pilot hole.
2. Soil is minimally de-structured around the sensor to avoid external influences on the measurements.

#### 2.2.1. Dimensioning

Electrodes and inter-electrodes must be large enough to ensure connectivity and minimize parasitic capacity between electrodes. Analytical calculations point to a diameter of 40 mm and electrodes spaced 10 mm apart to permit soil moisture detection.

**Figure 3:**
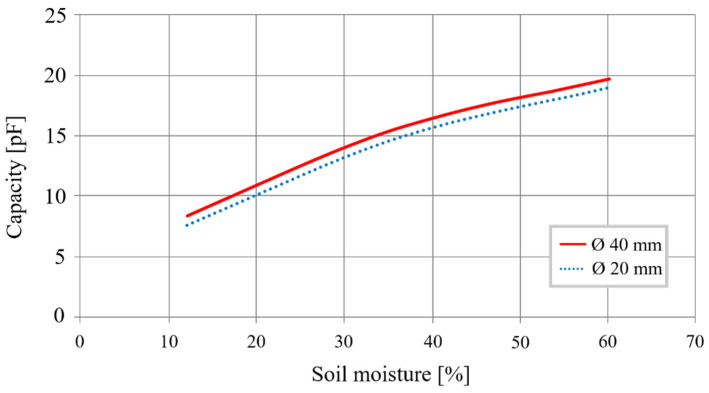
Different sized cylindrical sensor capacity as a function of moisture.

First, the surface of the electrodes must be limited to lower the cost of fabrication. The objective is to reduce the transducer’s diameter while preserving the homothetic dimensions relationship to affect the transducer’s ultimate capacity minimally. For re-dimension, the transducer makes it possible to obtain a 20 mm diameter on electrodes with a width of 30 mm, spaced 8 mm apart. Figure 3 shows the capacities evolution as a function of mass soil moisture for a diameter of 20 mm instead of 40 mm.

(2) $$Sensitivity=\;\frac{C_{60\%}-C_{12\%}}{60\%-12\%}$$

Curves show that the 20 mm transducer has the same characteristics as the transducer with a 40 mm diameter. Using Equation (2), we obtain a sensitivity of 0.26 pF/%, a variation of 12.6 pF after a 48% change in moisture.

**Figure 4:**
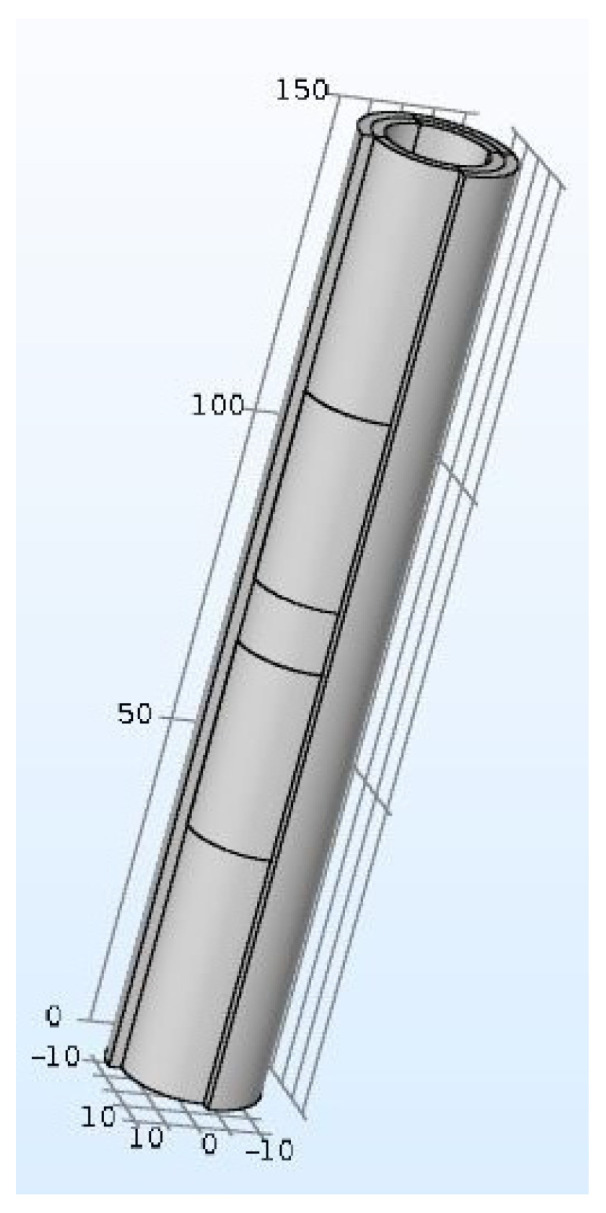
Cylindrical sensor shape.

**Figure 5:**
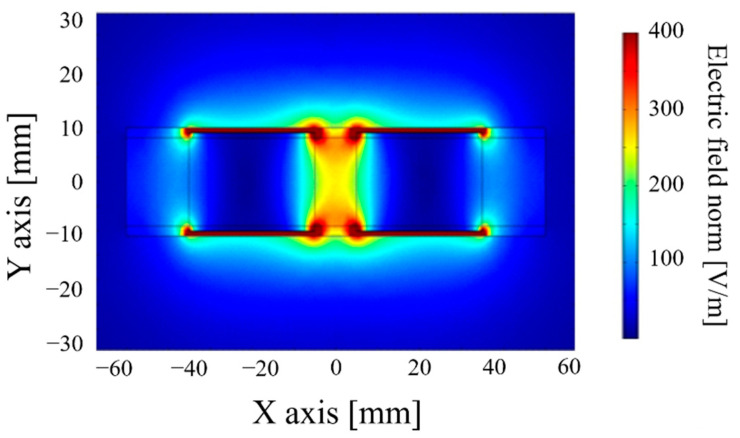
Electric density field for a 20 mm cylindrical sensor.

Beyond the sensitivity of the sensor, it is necessary to estimate the volume of the soil probed. COMSOL Multiphysics ^®^ carries out finite-element modeling. The soil probed volume is quantified based on the electric field’s density generated (Figure 4 and Figure 5). COMSOL can only give the capacity of the system in a defined soil volume. By varying the volume and recalculating the capacity, it is possible to determine the smallest detectable variation of capacity for the sensor electronics, and we can then obtain the volume associated with the capacity measured.

**Figure 6:**
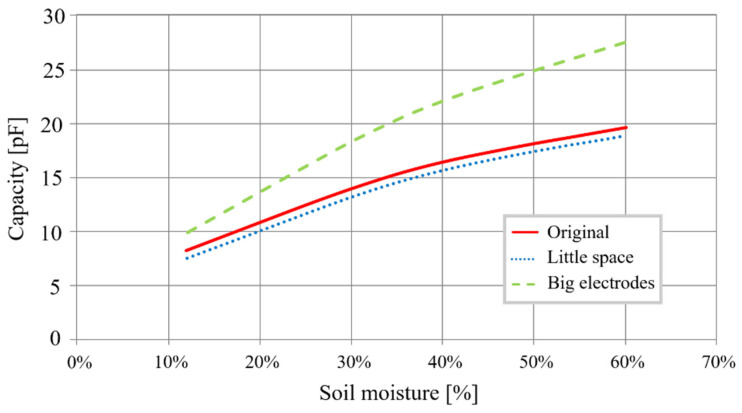
Different-sized electrodes capacitance as a function of moisture.

These simulation results show that a cylinder-shaped transducer with adapted electrodes (centered between (−20; 0) and (20; 0)) makes it possible to observe a 50 mm radius with an electric field density higher than 50V/m, which means that the soil volume is approximately 1.32 dm^3^. A sensitivity of 0.26 pF/% is needed to probe a sufficient volume of soil electronically. Yet, the copper surface needed is relatively significant (15,080 mm^2^), which raises the sensor’s cost. To optimize the dimensions of the transducer and thereby reduce the surface of the electrodes, Table 1 and Figure 6 present results obtained:

The analysis of these results allows us to exploit them according to the “rules of electrode design”:

1. If we reduce inter-electrode space, capacity is smaller, and sensitivity is reduced by 11%, from an initial 0.265 pF/% to 0.235 pF/%. This dimensional variation is therefore not viable. The augmentation of this space increases the size of the sensor and thus also its cost. It is preferable, therefore, to preserve a 10mm space between electrodes.
2. If we increase the electrode width, we increase the capacity as well as the sensitivity of the sensitive element. In fact, we go from 0.265 pF/% to 0.367 pF/%, an increase of 38%. However, we also increase the electrode surface by 67%, which raises its cost. The increase in sensitivity is not significant enough to justify the resulting cost increase.
3. If we reduce the electrodes’ width, we reduce sensitivity, but we lower the cost.

**Figure 7:**
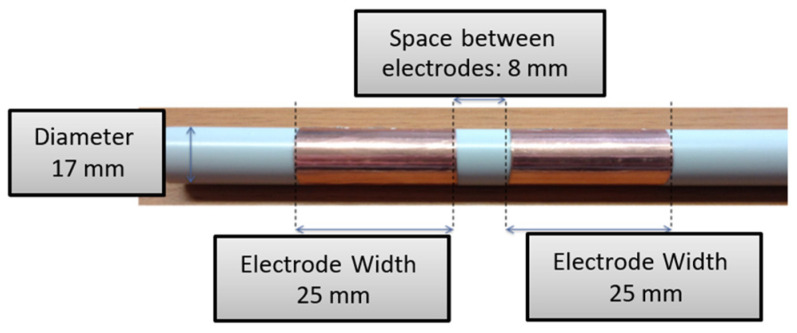
Sensor optimized design.

We use the Hadamard matrix to optimize the geometric sensor design. Figure 7 presents the sensor’s final shape equipped with cylindrical electrodes. The diameter is now reduced to 17 mm to accommodate the industrial process, which needs this reduction in order for the final sensor to have a 20 mm diameter.

**Figure 8:**
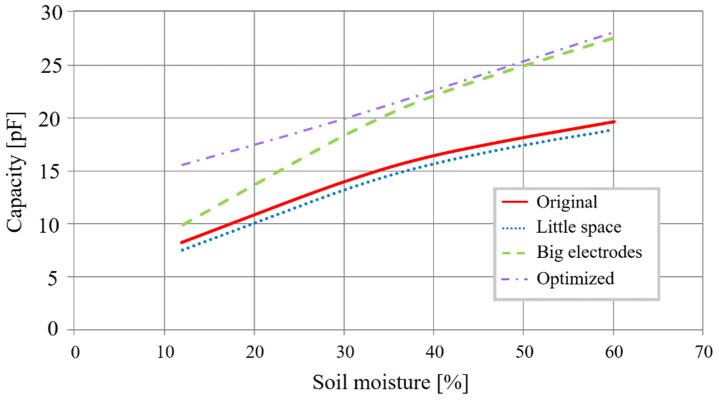
Comparison of the optimized design with the previous ones.

With this new design, we obtain a linear sensitivity and at a lower cost (Figure 8). The sensitivity is now at 0.2656 pF/%, and with a surface of 2670.35 mm^2^, the soil’s volume probed is about 1.13 dm^2^.

#### 2.2.2. Electrodes Materials Choice

**Figure 9:**
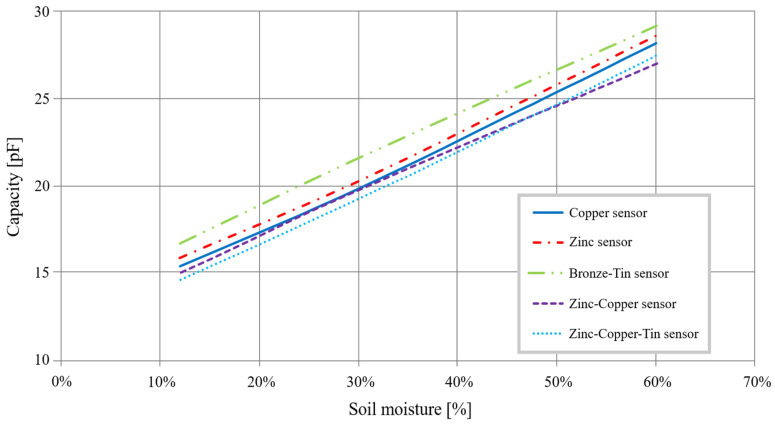
Different materials capacitance as a function of moisture.

As the geometric dimensions are now optimized, we want to evaluate less costly materials’ influence while preserving the same electric characteristics as copper. The identified materials are zinc, bronze, tin, or their alloys. To conduct this study, a partnership with the company Gilbert Polytech SAS made it possible to produce several prototypes which we evaluate with an impedance analyzer. It should be noted that, in the absence of the exact composition of alloys (for confidentiality reasons), we have not carried out simulations with the final elements. Figure 9 shows the measurement results obtained.

Figure 9 shows that copper, zinc, and zinc-bronze-tin are suitable for the fabrication of electrodes. Nevertheless, given the steam fabrication process, copper is the easiest to work with despite its higher cost. The deposit process is also found to be more reliable for the fabrication of the electrodes. Therefore, the transducer’s metal electrodes will be made of copper.

### 2.3. Toward New Shapes with Higher Performance

#### 2.3.1. New Sensor’s Shape

With the cylindrical shape optimized from a geometric and technological perspective, we can now focus on a complete optimization of the probed soil’s sensitivity and volume. To do so, we propose optimizing the electrodes’ shape factor.

**Figure 10:**
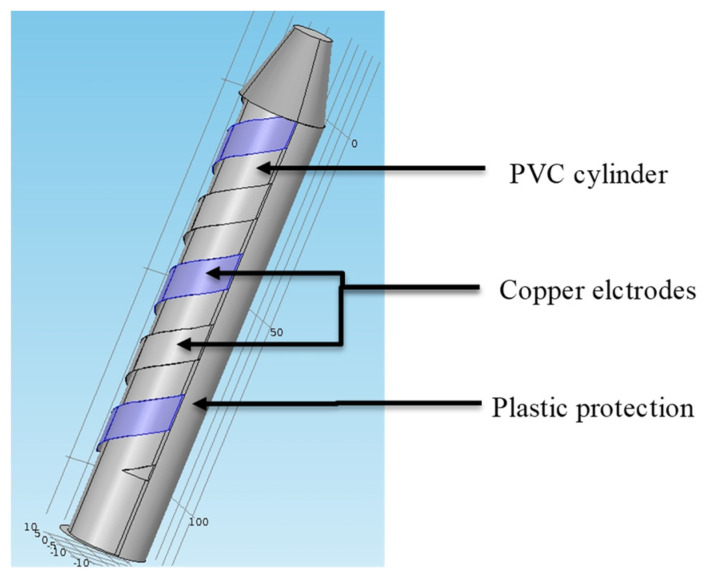
Helicoidal sensor shape.

**Figure 11:**
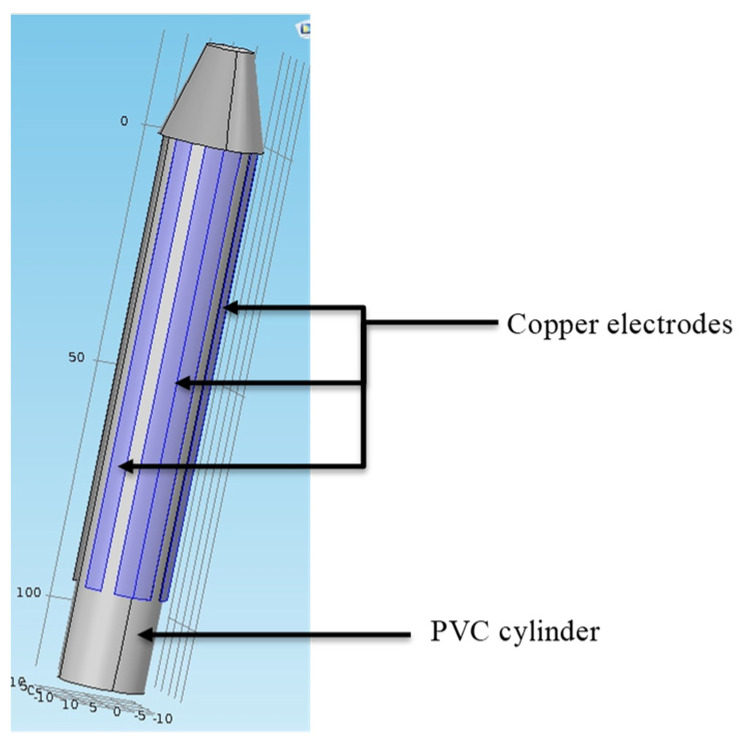
Branches sensor shape.

The first parameter to optimize is sensitivity. Knowing that this parameter depends on the contact surface between the soil and the electrodes, it is logical to increase the contact surface. However, in order to stay within initial cost limitations, the useful surface of the electrodes cannot be augmented. It must be distributed differently along the full length of the sensor. The conceptual idea that we defend consists of spreading the electrodes longitudinally around the cylinder by shaping them into a double helix (Figure 10). This approach is directly inspired by the strands of the Deoxyribonucleic acid (DNA). The second parameter to optimize is the volume of soil probed. Knowing that this parameter depends on the transducer’s field line paths, optimization depends on augmenting them. In practice (Figure 11), we distribute the electrodes in branch shapes on the cylinder’s periphery, placing them diametrically opposite each other.

The sensor with vertical ribbons (Figure 11) is equipped with two electrodes broken down into three strips, a main one in the center and two 110 mm lateral ones, for a total copper surface of 2670 mm^2^.

#### 2.3.2. Performance of These New Shapes

**Figure 12:**
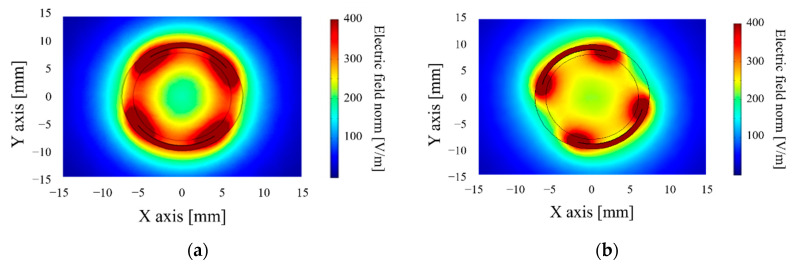
Electric field density around the first design (**a**) helicoidal sensors and the optimized design (**b**).

The simulation results confirm that the field created by the helicoidal sensor (Figure 12) is more uniform than that generated by the cylindrical shape. If the soil probed is traversed by an electric field of at least 50 V/m, the helicoidal transducer probes a volume of 0.165 dm^3^; after an optimization process, the probed volume is 0.277 dm^3^ compared to the 1.13 dm^3^ probed by the cylindrical shape. This decrease is after the contact surface division with a consequent reduction in these electric field paths. The volume probed is comparable with the Decagon sensor, which specifies a volume probed of 0.25 dm^3^ in its datasheet.

**Figure 13:**
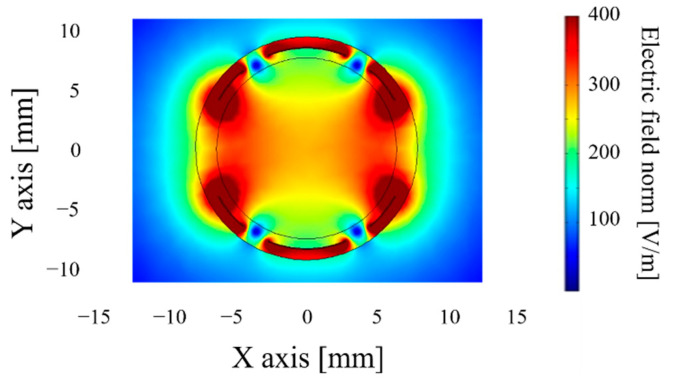
Electric field density around the sensor with branches.

As for the sensor with branches (Figure 13), its range of action is more significant than the preceding shape. The volume of soil probed reaches 1737 dm^3^, or six times more than the optimized helicoidal framework, as well as 1.5 times more than the initial structure (q.v. §3.1). Therefore, this is the new shape factor that permits soil testing on a larger scale.

**Figure 14:**
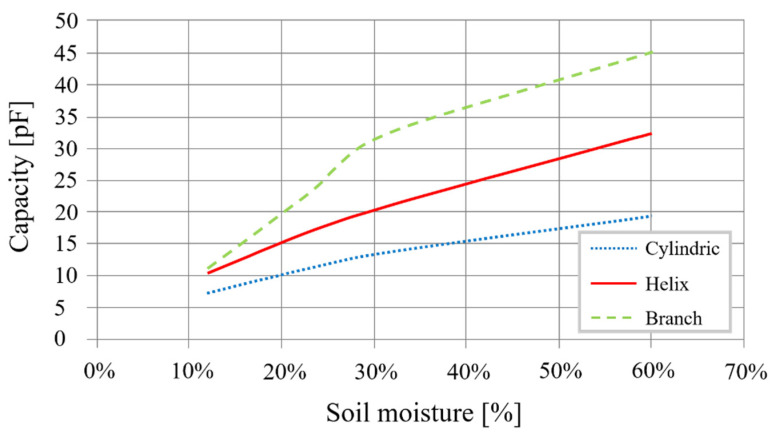
Different electrode shapes capacitance as a function of sensitivity to moisture.

To illustrate the evolution of electrical sensitivity, Figure 14 presents simulation results for the different shapes: cylindrical, double helix, and branches.

The double helix shape’s sensitivity is 67% higher, with a sensitivity of 0.4417 pF/% compared with the cylindrical sensor’s 0.265 pF/%. Moreover, response linearity is much better. The transducer obtained is, therefore, more sensitive for an equivalent cost. The sensor with branches also improves its sensitivity—by 0.7188 pF/% or 171%. Nevertheless, the response obtained is not linear throughout the entire measurement. The graphic (Figure 14) shows two linear zones before and after 27% moisture in the soil. The electronic treatment will consequently require two reading equations.

#### 2.3.3. Choice of the Final Shape

Table 2 reflects the characteristics of the three sensors studied. The overall performance of the sensor with branches stands out.

The sensor with branches is not only more sensitive, it also registers a higher soil volume. Its response is not linear, but the graphic highlights two linear zones that can be converted into two equations, which can be selected using a comparator. This shape is, therefore, the most suitable for our study. Nevertheless, this shape is also the most expensive to industrialize due to the electrodes’ discontinuous character. Manufacturers agree that the cost of fabrication is two times higher compared to the cylindrical or helicoidal shapes. Economic considerations, therefore, prevail over performance in the choice of a helicoidal shape for the sensor.

### 2.4. Modeling the Helicoidal-Shaped Sensor

#### 2.4.1. Frequency Analysis

**Figure 15:**
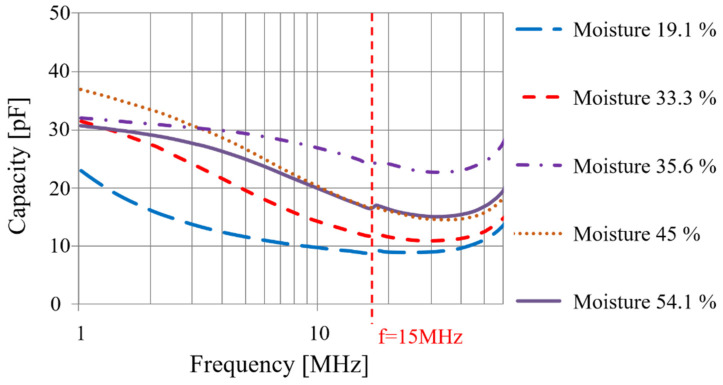
Measured capacitance as a function of the frequency.

Given the sensor’s macroscopic capacitive behavior as a function of moisture in the soil, this analysis (Figure 15) is necessary to identify the useful measurement frequency ranges.

Figure 15 shows that:

1. Capacity variations are inversely proportionate to moisture in the soil, which confirms the theoretical functioning.
2. As expected, the capacity is not fixed depending on the frequency, but the more the frequency increases, the more the capacity diminishes until it reaches approximately 15 MHz.
3. Beyond this frequency, the inductive parasitic effect is no longer negligible. This demonstrates that beyond 15 MHz, it becomes difficult to devise an exploitable electronic conditioner as measurements are carried out below this frequency.

Since most of the significant variances in capacity, as a moisture function, fall between 1 MHz and 15 MHz, a working frequency close to 8 MHz is chosen for the electronic conditioner.

**Figure 16:**
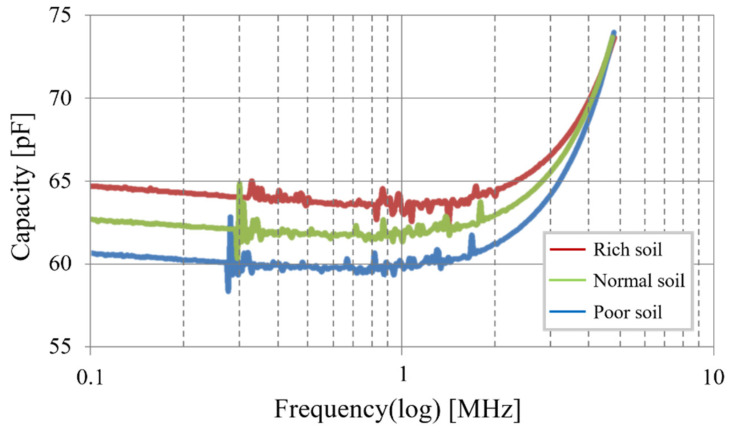
Sensor capacitance as a function of the frequency for different levels of salinity.

We aim to determine if it would now be possible to endow the sensor with a second aptitude so that it could measure salinity in the soil, depending on the possibility of finding a second frequency interval that would be sensitive to variations in soil salinity. To this end, we install a measurement protocol analogous to the moisture measurement. Capacity is measured within a frequency ranging from one hundred kilohertz to around ten megahertz (Figure 16) with three different soils, one with no fertilizer (Poor soil), one with a fertilizer saturation (Rich soil), and one between these two (Normal soil).

**Figure 17:**
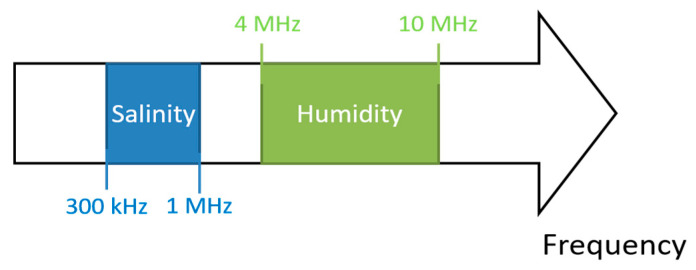
Dedicated frequency ranges for single shape sensor (salinity or humidity).

We observe that for frequencies higher than 4 MHz, salinity does not influence the sensor’s capacity. This means that the sensor’s moisture measurement is not affected by the soil’s salinity, making the measurement independent. But below 4 MHz, capacity increases with salinity. Yet, capacity does not depend on the measurement frequency as much as it does for the moisture measurement. There is no preferred zone in this curve, but perturbations are observed below 300 kHz, making it necessary to avoid this zone. It is therefore possible to take measurements on all frequencies between 300 kHz and 1 MHz. Figure 17 summarizes all useable measurement frequencies.

#### 2.4.2. Coating Effect

**Figure 18:**
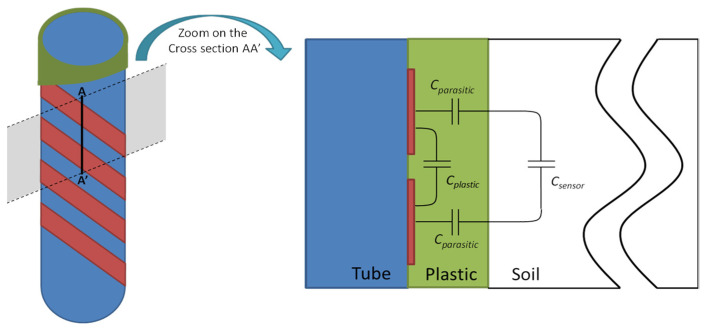
Coating protection parasitic effect.

For mechanical protection reasons, we have studied the effect of plastic coating on the sensor. Figure 18 provides a zoomed-in view of a cross-section of the sensor. This cross-section localizes the coupling capacity between electrodes, coating, and soil.

Two supplementary pathways generated by the molding can be observed:

1. First, field lines close to the sensor only traverse the plastic and not the soil. This creates parasitic capacity wherever the dielectric is in the plastic coating. Since this plastic is hermetic, its parameters are independent of moisture.
2. Second, far-off field lines traverse the soil and traverse a layer of plastic coating, which produces a series of parasitic capacity in addition to soil measurement capacity.

**Figure 19:**
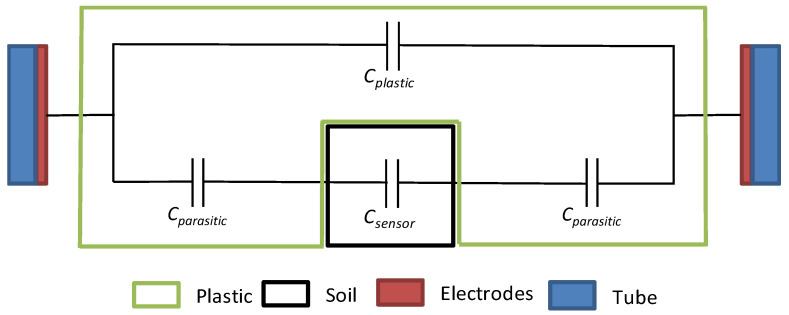
Sensor’s equivalent model considering his coating protection.

The transducer system may then be modeled as coated, according to the equivalent electrical diagram presented in Figure 19.

This model reveals two parasitic effects:

Parallel *C_plastic_* capacity that adds to the *C_humidity_* measurement capacity.

A *C_humidity_* measurement capacity is altered by two parasitic capacities *C_parasite_*. which result in in serial association with capacity *C_parasite_*/2. This new measurement capacity is found to be inferior to that of a non-integrated sensor.

**Figure 20:**
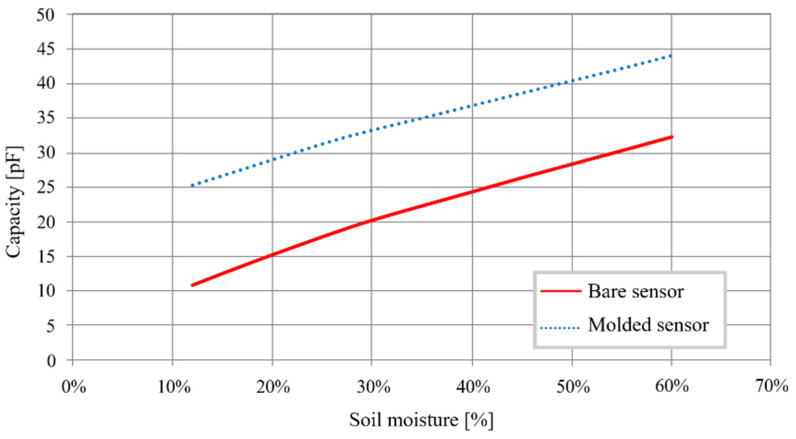
Molded and bare sensor capacitance as a function of humidity.

Figure 20 shows the influence of the molding on the sensitivity of the sensor.

Two effects on the preceding model can be observed:

1. The curve of the molded transducer is higher at every point. It reflects the increase in C’_hum0_, estimated at 14.7 pF.
2. Sensitivity is reduced by 12%, with an estimated value of 0.3875 pF/%. This is measurable by our system with a relative variation of 1% in moisture.

It should be recalled that the salinity measurement is affected by the same precise order of grandeur as that of moisture.

#### 2.4.3. Equivalent Sensors’ Electrical Models

**Figure 21:**
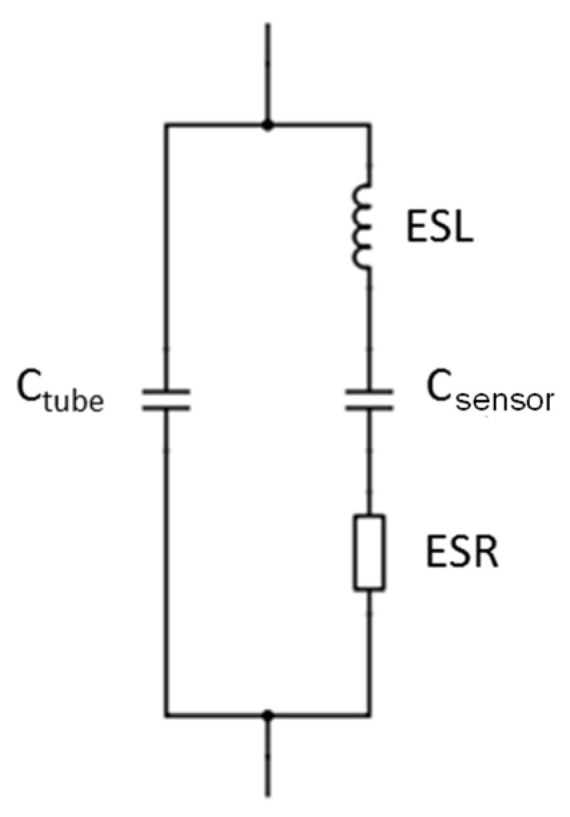
Sensor’s electrical model.

Since sensor capacity is not perfect, we want to quantify the ESR, ESL, and *C_electrodes_* imperfections of the equivalent model. The Equivalent Series Resistor (ESR) is the resistance that materializes in contact between the two electrodes, with a measured value of ESR = 105 mΩ. Equivalent Series Inductance (ESL) consists of the connecting wires that induce the inductive behavior beyond 40 MHz. Measurements show that ESL = 60 µH. Finally, inter-electrode capacity is equal to *C_electrodes_* = 5.18 fF. Figure 21 reflects this model.

**Figure 22:**
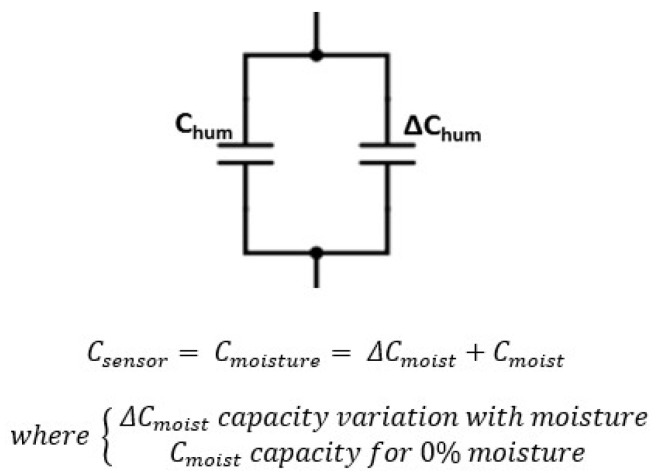
Sensor final electrical model as a function of moisture.

**Figure 23:**
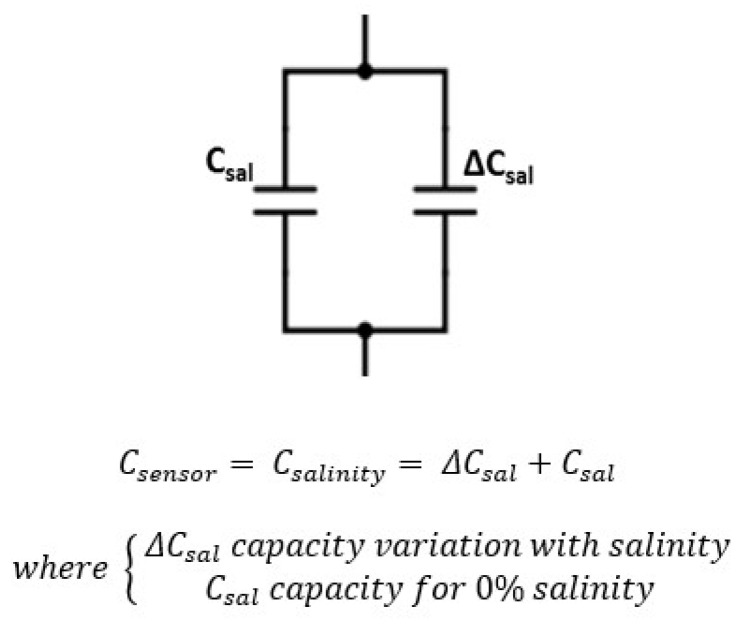
Sensor final electric model as a function of salinity.

*C_sensor_* capacity is associated with the type of measurement (moisture or salinity). Equivalent models to its capacity are shown in Figure 22 and Figure 23.

**Figure 24:**
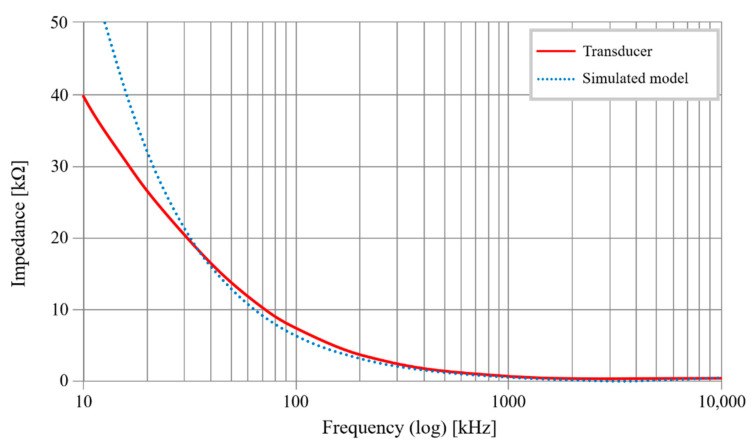
Sensor model: simulated vs. transducer results.

An OrCAD PSpice simulation makes it possible to check the transducer’s experimental results against the electric model. The behavioral results are presented in Figure 24.

With an impedance of 2.3 kΩ at frequency f = 35 kHz, the curves are almost the same; then, as of f = 400 kHz, the curves are identical. Since useful frequency ranges are well above f = 35 kHz, the sensor’s electric model is validated.

### 2.5. Electronics Measurements

#### 2.5.1. Architecture

**Figure 25:**
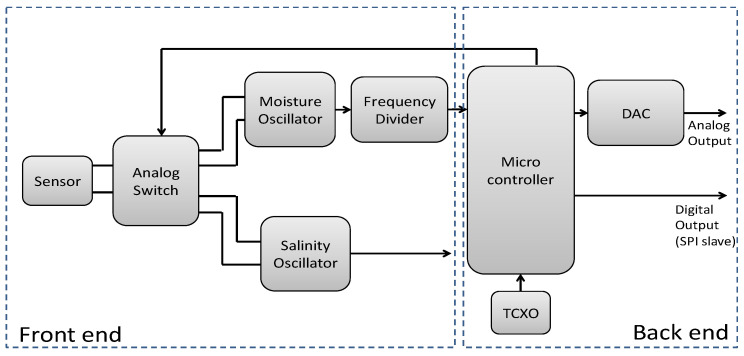
Functional diagram of electronics measurement.

The architecture developed breaks down into two distinct parts (Figure 25). The first part is the front end which permits the conversion of the sensor’s capacity variations into one electric variation that is exploitable. Bearing in mind the frequency of readings and the final system’s low cost, the sensor is placed in a Colpitts oscillator to allow a capacity conversion frequency that depends on moisture or salinity. Since the sensor must perform two measurements, we have decided to use two different oscillators set at f_osc_ = 8 MHz for moisture and f_osc_ = 500 kHz for salinity.

The back end, or the second part of the architecture, delivers readings of the variations. The back end is structured around one microcontroller embedded with a frequency meter algorithm dedicated to double measurement of a frequency. The microcontroller is equipped with a TCXO-type precision clock to minimize the temperature effect of the measure. This microcontroller’s frequency performance is voluntarily limited to reduce the system’s cost, this explains why the frequency is divided. Once this electronic treatment is accomplished, the back end proposes two complementary outlets, an analog outlet that delivers voltage proportional to moisture or salinity with an ADC aid, and a digital outlet that can be configured to establish a communication link with a standardized protocol (slave SPI). We have decided to implement two outputs, one analogic and another digital. This gives the choice to use another microcontroller to recover and process the digital output or implement an analogic instrumentation chain.

#### 2.5.2. Material Integration

**Figure 26:**
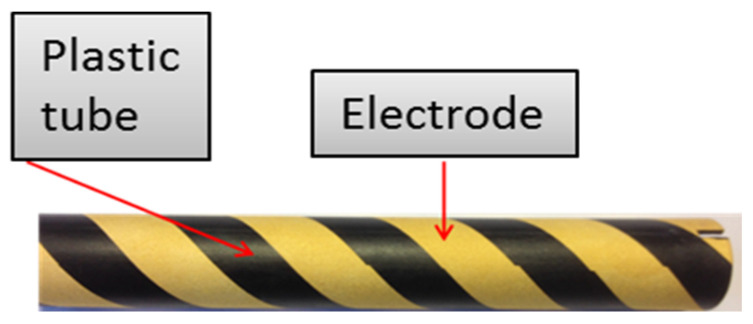
Industrial sensor’s fabrication.

The sensor tube has been manufactured according to an industrial procedure. The first stage consists of devising a cylindrical support tube. Electrodes are then produced according to photolithography and by an electrochemical copper’s deposit on a surface made functional by a laser. Finally, a nickel/gold layer is deposited to protect the copper from oxidation. Figure 26 presents the finished sensor produced by this process.

**Figure 27:**
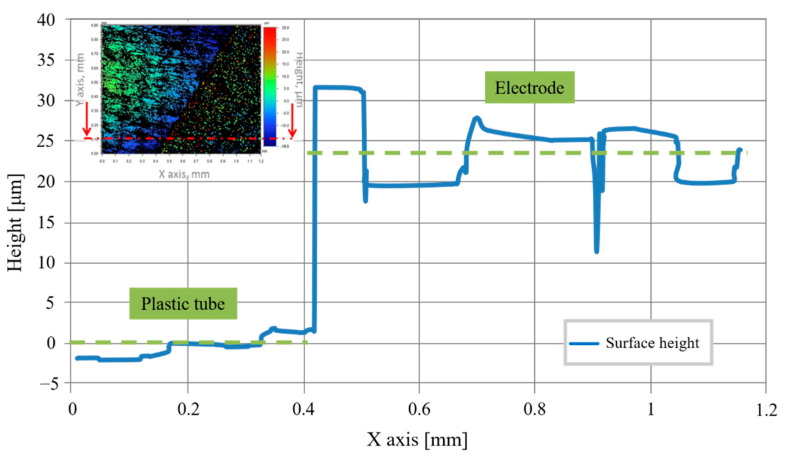
Thickness measurement.

The electrode’s electrical conductivity is ensured by controlling the metallic deposit thickness measured by an optical profilometer (Figure 27). The sensor’s metallic thickness layer presents a value of about 25 µm.

This figure shows:

1. On the left of the graphic, we can see the plastic tubing. It serves as a reference to measure the height of the electrodes.
2. On the right, the measurement of the metallic deposit’s average height: 24 µm (20 µm Cu, 4 µm NiAu) is illustrated.

**Figure 28:**
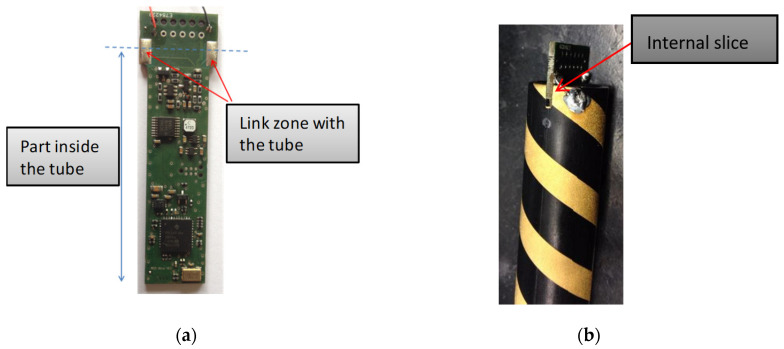
(**a**) PCBA embedded electronic measurement; (**b**) Interconnection between PCBA and the sensor tube.

Since the sensor is inserted in the soil, it needs to be mechanically solid so that it is not damaged during installation. The electronic board (Figure 28a) is inserted in the sensor tube to form a compact unit with no flexion points. To promote the ensemble’s mechanical longevity, the recesses (Figure 28b) have been specifically sized.

**Figure 29:**
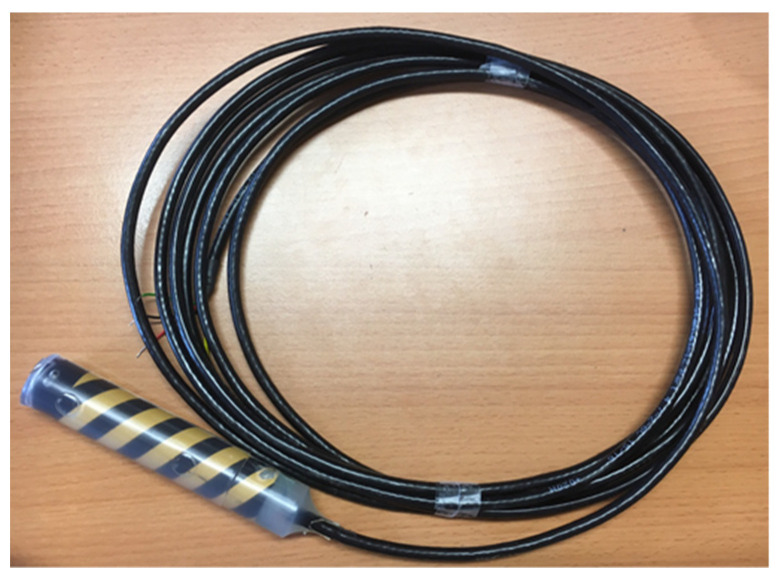
Industrially molded sensor.

A protective shield is directly soldered onto specific landing pads to permit power supply and communication with the system. Finally, the sensor’s water tightness is ensured by an injection of plastic into a mold. Figure 29 is a photo of the final sensor.

#### 2.5.3. From the Sensor to the Final User

**Figure 30:**
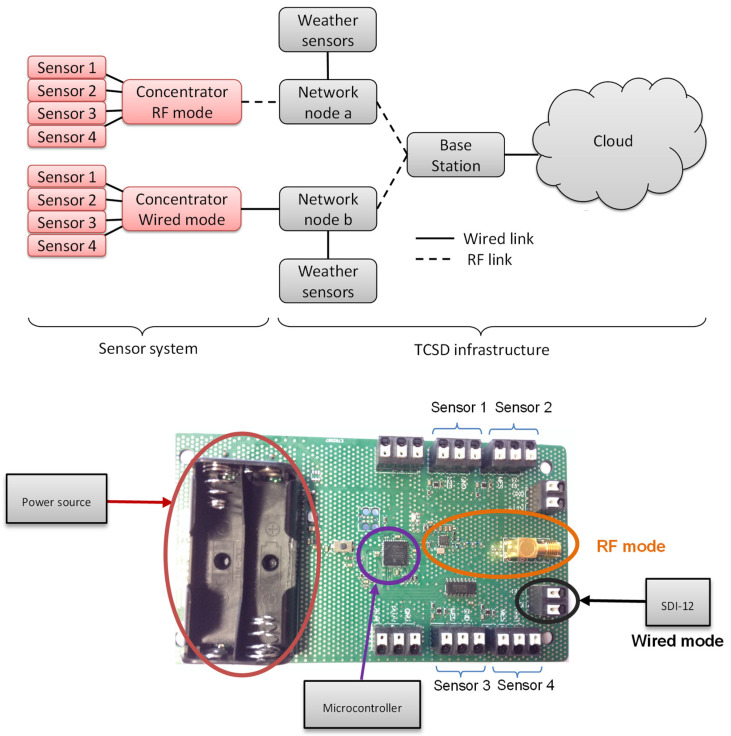
The architecture of the sensor network and the PCBA associated.

To deploy the sensors in cultivated fields, the network architecture is shown in Figure 30. This infrastructure makes it possible to ensure the transmission of moisture and salinity measurement readings to the user, and was developed with the support of TCSD, a company which markets data-collection systems for agriculture (weather and soil).

Figure 30 also shows the concentrator architecture:

1. A concentrator supervises each sensor. Each concentrator can collect data from four sensors. It then communicates this data by a wire connection with a specific SDI-12 protocol, either by a radiofrequency link on the ISM 868 MHz band to allow total control of the exchange protocol. Tests demonstrate a radio range of 600 m with a baud rate of 9600 bits/s. The concentrator’s energy autonomy is provided by four AA batteries that last for an entire irrigation season (about 8 months). Figure 30 shows the electronic board.
2. Data then goes through the other network nodes that could be concentrators used in repeating mode or weather data collection points that also could serve as the bridge.
3. Data finally arrives at a base station connected to the Internet, where information is stored on an online server. From then on, the user can consult the data through a web interface or mobile application.

### 2.6. Experimental Results on Site

**Figure 31:**
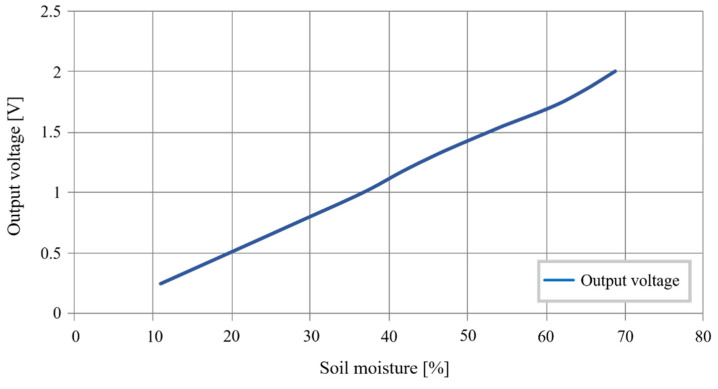
Sensor’s output voltage as a function of the soil moisture.

First, laboratory tests are carried out to validate the sensor’s behavior when measuring moisture and salinity. Figure 31 shows the sensor’s output voltage responding to the soil moisture. These measurements present a variance of 0.6 V/%, standard deviation of 0.36 V/%, and linearity of 93.33%.

We observe that the following linear relation can approach the data obtained:

(3) $$V_{s}\left(V\right)=0.0301*RH\left(\%\right)-0.0898$$

These tests prove the feasibility of the soil moisture measurement.

**Figure 32:**
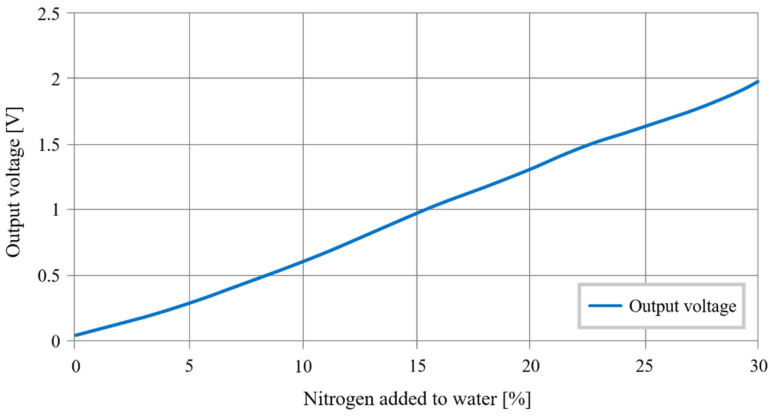
Sensor’s output voltage as a function of the soil salinity.

Then, Figure 32 shows the sensor output voltage responding to the salinity variations. To obtain these variations, soil samples are moistened to the same humidity, and nitrogen and common fertilizer are dissolved to modify soil salinity. These measurements present a variance of 0.56 V/%, a standard deviation of 0.31 V/%, and linearity of 98.27%.

We observe that the following linear relation can approach the data obtained:

(4) $$V_{s}\left(V\right)=0.0657*Nitrogen\;added\left(\%\right)-0.0023$$

The sensor does not have to be very precise. The farmers set a threshold below which it is needed to add fertilizer. The threshold changes with the type of agriculture. These tests prove the feasibility of the soil salinity measurement.

During these tests, a constraint appears. The soil around the sensor needs to be without air cavities. Indeed, the presence of air distorts the measurement, and the sensor shows false values. To avoid this problem in the field, we choose to bore a bigger hole than the sensor’s diameter and then pack the soil around it.

### 2.7. Longitudinal Moisture Measurements

After the system is installed and deployed in cultivated and irrigated fields, moisture monitoring performance is compared to two major players in the market: Decagon (Meter Environment, Munich, Germany) and Sentek Technologies (Sentek, Stepney South, Australia). As this work continues, the sensor that we have developed is called IRRIS for IRRigation Ingénierie Systèmes.

**Figure 33:**
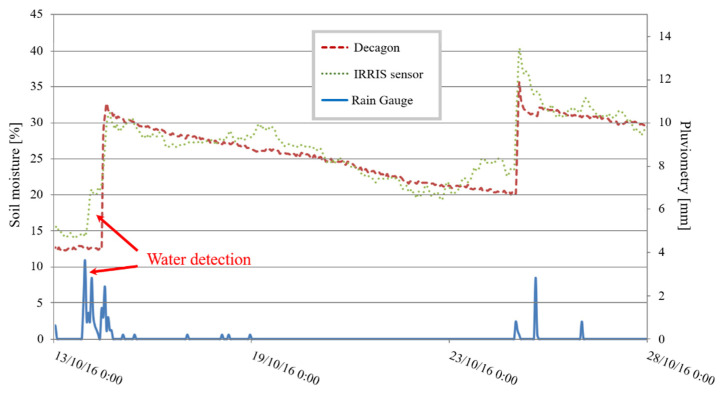
Sensors’ tests in an orchard.

Figure 33 presents measurement readings in an orchard that is cultivated all year round. Data from the IRRIS sensor shown on the graphic reflects three weeks of surveillance and is compared with that of the Decagon sensor EC-5. The sensors are placed at a depth of 10 cm.

The higher sensitivity of the IRRIS sensor is noticeable; the moisture measurement increases as soon as water is detected by the IRRIS sensor. In contrast, the Decagon sensor does not detect this water presence until after a few hours (red arrows). Detection, therefore, is better with the IRRIS sensor. The rising dynamic (+20%) and the descending dynamic (−2% per day) are identical for the two sensors, validating the sensor’s good performance on this piece of agricultural land.

**Figure 34:**
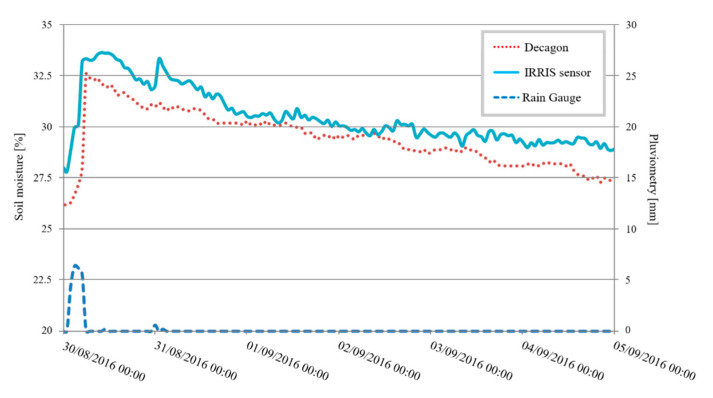
Sensors’ tests in a cornfield.

The second study addresses the surveillance of a cornfield. The measurement readings reflect several continuous surveillance days because of an installation made at the end of a cultivation cycle before the lot is harvested. Figure 34 presents the results obtained. The sensors are placed at a depth of 30 cm.

This figure compares the dynamic and the response time of the IRRIS sensor compared with the Decagon sensor. In terms of reactivity, the IRRIS sensor reacts 30 min before the Decagon’s. As regards dynamics, the moisture measurement rises due to the identical water supply for both sensors (+7%). The descent dynamic is more rapid for the Decagon since it has been placed closer to the plants, and water is therefore more rapidly absorbed by the plants. These measurement readings testify to the good performance of the IRRIS sensor on this type of crop.

**Figure 35:**
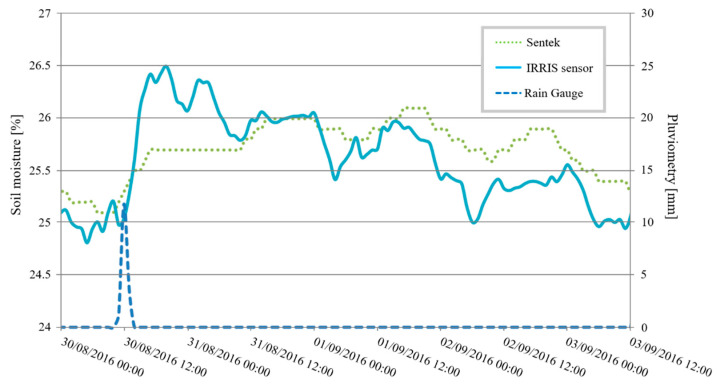
Comparison of the Sentek and IRRIS sensors in a cornfield.

The last test is conducted in another cornfield with a Sentek sensor. Figure 35 presents the results.The sensors are placed at a depth of 10 cm.

The IRRIS sensor dynamics resemble those of the Sentek’s. A rise in moisture is measured after a water supply, followed by a slow descent on the scale. Moreover, this graphic shows an oscillation effect in the soil during a day/night cycle. The IRRIS sensor also measures this phenomenon, which validates our sensor’s performance.

To conclude, these tests have shown that comparative response curves for the IRRIS sensors and the commercially available sensors attest to the superior performance of the IRRIS sensor. In this paper, only the sensor near the surface has been exhibited because there are no variations deeper in the soil. This paper has shown that it is possible to detect small amounts of water more rapidly. This work clearly demonstrates the possibility of following these dynamic variations of moisture in the soil, when water is being supplied, and when the soil is drying out due to lack of water.

## 3. Conclusions

First, this study presents the methodology to measure soil moisture and salinity. Currently, the best fit for the farmers’ expectations is the capacitive method. It allows them to have a quick response time with reliable accuracy. However, the existing systems, with their prices or complexity, do not allow for a greater deployment. This paper presents the development of a new capacitive sensor that can multiply the points of measure in the fields.

Subsequently, the sensor’s shape is designed. Based on a capacitor with two electrodes and a dielectric, a cylindric shape was optimized. The sensor has a sensitivity of 0.265 pF/% for a probed soil’s volume of 1.32 dm^3^. New innovative electrode shapes were tested. These shapes improve either the sensitivity or the volume probed. Finally, the helix shape was chosen to obtain a 0.4417 pF/% sensitivity despite the probed soil’s volume, which is 0.277 dm^3^.

Next, the electronics are conceived. It is composed of two parts. The first one, the front end, is based on a Colpitts oscillator that transforms the capacitive variation into a frequency variation. The second one, the motherboard, is designed around an embedded intelligence that reads the frequency and interacts with a wired connection that can be analog or digital. A concentrator on the ground allows the system to be autonomous by providing an 868 MHz radio link and batteries’ power supply.

Finally, two types of tests are implemented. The first one is carried out in the laboratory by reproducing different soil moisture and salinity. This feasibility study proves the sensor’s ability to take the aforementioned measurements. On-site tests validate the sensor’s soil moisture sensing. In future studies, tests will be carried out to validate the salinity sensing in the field and the hysteresis of the sensor.

To conclude, the sensor performs better than commercially available products and has other benefits, one being the shape design. The short cylindrical shape can be inserted easily in the soil without destroying the surrounding soil. Moreover, it has different outlets (analogous wire, digital with or without wire), offering a direct connection to most existing systems.

Furthermore, with a lower cost of fabrication than other commercially available products (estimated at less than 50 € per unit), its price makes it possible to multiply measuring points to better chart the hydric state of cultivated soil, which is its main goal. 

## Figures and Tables

**Table 1:** New electrode dimensions.

Sensor	Diameter (mm)	Space between Electrodes (mm)	Electrodes Width (mm)	Electrodes Surface (mm^2^)
Original	20	10	30	3769.91
Little space	20	5	30	3769.91
Big electrode	20	10	50	6283.19

**Table 2:** Summary of the three sensor’s characteristics.

Electrode Shape	Sensitivity (pF/%)	Linear Response	Probed Soil (dm^3^)	Copper Surface (mm^2^)
Cylindric	0.265	Yes	1.32	2670.35
Double spiral	0.4417	Yes	0.277	2670.35
Branches	0.7188	No	1.737	2670.35
